# Caring for Caregivers (C4C): study protocol for a pilot feasibility randomised control trial of Positive Written Disclosure for older adult caregivers of people with psychosis

**DOI:** 10.1186/s40814-017-0206-z

**Published:** 2017-11-21

**Authors:** Cassie M. Hazell, Christina J. Jones, Mark Hayward, Stephen A. Bremner, Daryl B. O’Connor, Vanessa Pinfold, Helen E. Smith

**Affiliations:** 1 Department of Primary Care and Public Health, Brighton and Sussex Medical School, Mayfield House, Village Way, Falmer, BN1 9PH UK; 2 Department of Clinical Medicine, Royal Alexandra Children’s Hospital, University of Sussex, Eastern Road, Brighton, BN2 5BE UK; 3 Sussex Partnership NHS Foundation Trust, Research and Development Department, Sussex Education Centre, Nevill Avenue, Hove, BN3 7HZ UK; 4 School of Psychology, University of Leeds, Leeds, LS2 9JT UK; 5 Family Medicine and Primary Care, Lee Kong Chian School of Medicine, Nanyang Technological University, Novena Campus, 11 Mandalay Road, Singapore, 308232 Singapore; 6 The McPin Foundation, 32-36 Loman Street, London, SE1 0EH UK

**Keywords:** Carer, Caregiver, Written emotional disclosure, Positive written disclosure, Psychosis, RCT, Older adult

## Abstract

**Background:**

The caregivers of people who experience psychosis are themselves at risk of developing physical and mental health problems. This risk is increased for older adult caregivers who also have to manage the lifestyle and health changes associated with ageing. As a consequence, older adult caregivers are in particular need of support; we propose a Written Emotional Disclosure (WED) intervention, called Positive Written Disclosure (PWD).

**Methods/design:**

This is a pilot randomised controlled trial of PWD compared to a neutral writing control and a no writing condition. We aim to recruit 60 participants, 20 in each arm. This study will utilise a mixed-methods approach and collect quantitative (questionnaires) and qualitative (interviews) data. Quantitative data will be collected at baseline and 1, 3, and 6 months post baseline. Participants who complete a writing task (PWD or neutral writing control) will be invited to complete an exit interview to discuss their experiences of the intervention and study. The study is supported by a patient and public involvement group.

**Discussion:**

The results of this trial will determine whether a definitive trial is justified. If so, the quantitative and qualitative findings will be used to refine the intervention and study protocols.

**Trial registration:**

ISRCTN, ISRCTN79116352. Registered on 23 January 2017

**Electronic supplementary material:**

The online version of this article (10.1186/s40814-017-0206-z) contains supplementary material, which is available to authorized users.

## Background

Many people who experience psychosis require occasional or regular support from family or friends to complete their activities of daily living, and for some, this support is needed on a longer-term basis [1]. The family and friends that provide this support are generally referred to by clinical services as caregivers [2]. The role of these caregivers is vital for both the care recipient and society as a whole. Caregivers of people with psychosis spend on average 5.6 h a day providing care—this equates to more hours per week than a full-time job [3]. In the UK, it would cost the National Health Service (NHS) £34,000 per annum to hire a support worker to provide the same amount of care [3].

Being a caregiver for someone experiencing psychosis can be physically, mentally, and emotionally demanding. These caregivers are at risk of developing a number of health problems, such as exhaustion [4], anxiety and depression [5], and posttraumatic stress disorder (PTSD) [6]. Many caregivers find it difficult to engage in leisure or social activities and maintain any form of paid employment [7]. For older caregivers, this risk is increased further as they have to manage their own age-related health difficulties alongside the challenges of caregiving [8, 9]. Despite these needs, healthcare services consistently fail to consider or provide support for caregivers [10].

The UK’s National Institute for Health and Care Excellence (NICE) [11] guidelines recommend that family therapy should be offered to everyone with psychosis and their families. Unfortunately, family medicine implementation rates continue to be poor [1, 12] due to limited resources and lack of service funding [13]. Instead, some clinical and third sector organisations offer caregivers support groups. While some caregivers report benefits from attending these groups, they do not suit others and the dropout rates tend to be high [14]. Caregivers report finding it difficult to make the time to attend (because of caregiving duties) and feeling uncomfortable talking in a group [15]. Alternative approaches to improve the wellbeing of caregivers need to be considered—particularly the ones that do not require significant clinical resources and are both acceptable and accessible for caregivers.

One possible intervention that could be helpful for these caregivers is Written Emotional Disclosure (WED). WED is a self-directed writing therapy that typically involves writing about a stressful or traumatic experience continuously for 20 min each time, for three consecutive days [16]. Multiple meta-analyses have demonstrated WED to be effective on a range of outcomes in both clinical and nonclinical samples [17–20], including small beneficial effects for caregivers generally [21]. However, very limited benefits have been found in trials of WED for caregivers of people with psychosis [22, 23].

An alternative form of therapeutic writing, called Positive Written Disclosure (PWD), has recently been evaluated in the research setting. PWD follows an identical format to WED, but the instructions ask participants to write about positive, rather than traumatic, experiences. A recent large, three-arm trial (comparing PWD to WED and a writing control task) showed that for some caregivers, PWD led to decreased anxiety and depression post-intervention and subsequent follow-up, whereas the caregivers undertaking WED experienced no improvements [24]. Although these findings are promising, they cannot automatically be extrapolated to caregivers supporting people with psychosis because of the unique and additional challenges—including the stigma of mental health problems and its impact on the wellbeing of the caregiver and their care recipient [25].

The long-term aim of this research program is to explore the effects of PWD for older adult caregivers of people with psychosis. The first step in this research program is to conduct a pilot feasibility randomised controlled trial.

The aims of the pilot trial are to (1) determine whether a definitive trial of PWD on older adult caregivers of people experiencing psychosis is justified in terms of recruitment, retention, reasons for dropout, and adherence to writing task; (2) estimate the pooled standard deviation for the primary outcome (mood) to be used for future sample size calculations in the event of a definitive trial being justified and gain insight into what an appropriate Minimal Clinically Important Difference (MCID) might be for future studies; and (3) identify whether PWD and the study design is acceptable, accessible, and feasible to participants by using a combination of questionnaires, recruitment and retention rates (quantitative), and post-study interviews (qualitative), all supported by a patient and public involvement reference group.

## Methods/design

This study is a single blind, external pilot randomised controlled trial (RCT) with three parallel arms: (1) Positive Written Disclosure (PWD), (2) Writing Control task (WC), and (3) Non-Writing Control group (NWC). Prior to recruitment, an independent statistician will generate a randomised group allocation sequence, allocating each participant ID to one of the three arms. The randomisation will use a 1:1:1 ratio block randomisation to create a randomly permuted sequence; this sequence will be concealed from the research team. The group allocation will be concealed from participants until they have completed their baseline assessment; after the baseline assessment, participants will receive an opaque, sealed envelope that contains their group allocation. The trial research assistants will remain blinded to participants’ group allocations throughout the study.

**Fig. 1:**
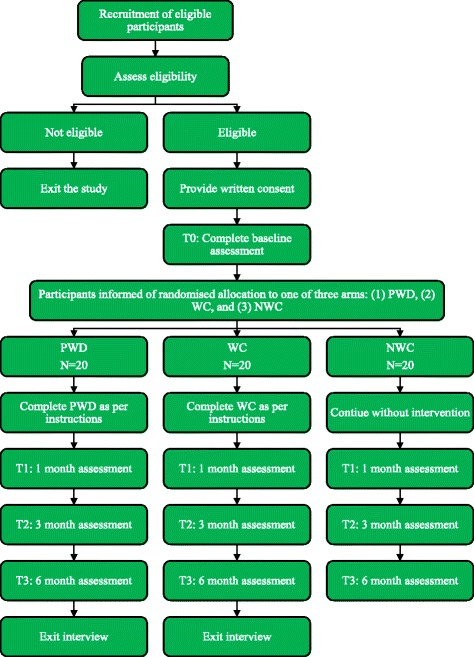
The C4C Study CONSORT. *Note:* PWD Positive Written Disclosure, WC Writing Control task, NWC Non-Writing Control group

We will collect the quantitative outcome data at baseline (T0—prior to participants being informed of their group allocation) and 1 (T1), 3 (T2), and 6 (T3) months post baseline. Participants will provide consent and complete the baseline assessment with a research assistant. Participants randomised to complete a writing task (either PWD or WC) will be asked to complete the writing task within a week of completing the T0 assessment. They will be asked to return their writing packs to the research team when they complete the T1 assessment. Participants will be able to request their writing back at the end of the study. All participants who are allocated to either the PWD or WC groups will also be invited to complete an exit interview after completing the 6-month assessment. See Fig. 1 for CONSORT diagram.

Carers of people with psychosis have been involved in each part of the research process. Their feedback has helped to shape this research study from its conception, through to design and delivery (see “Public and patient involvement” section for more information).

### Participants

Participants will be recruited using a number of routes, including via NHS services and third sector organisations. Mental health practitioners working in NHS secondary mental health services will be asked to identify the caregivers of anyone on their current case load experiencing psychosis who may be interested in this study. Also, local general practitioners (GP) will be asked to screen their carers register and patient records to identify any potential participants who meet the study criteria. Invitations will also be sent to any caregivers listed on our database of people who have consented to being contacted about research studies, and the research team will publicise the study through relevant local third sector organisations. We will be accepting both practitioner- and self-referrals. We plan to recruit a total of 60 participants (20 in each arm). This recruitment target is in line with the recommendations of Julious [26] that a minimum of 12 participants per arm is an appropriate sample size for pilot trials. Recruiting 20 participants per arm allows for a potential attrition rate of up to 40%.

The study will use the following inclusion criteria: participants must (1) be classified as a primary caregiver, as defined by the Royal College of General Practitioners and The Princess Royal Trust for Carers [2] (this includes “any person who provides unpaid support to a partner, child, relative or friend who couldn’t manage to live independently or whose health or wellbeing would deteriorate without this help” (p. 9)); (2) aged 60 years or over; (3) provide care for someone experiencing psychosis (this includes persons with a diagnosis of schizophrenia, schizoaffective disorder, schizotypal personality disorder, delusional disorder, psychosis not otherwise specified, bipolar, and depression with psychotic features); and (4) be able to read and write in English. Participants will be excluded if they are currently receiving psychological therapy, including family therapy, or if such therapy is scheduled to start in the next 6 months.

### Positive Written Disclosure (PWD)

The PWD intervention protocol will broadly follow the original Pennebaker and Beall [27] protocol, with the exception of the writing topic. Participants will be asked to write about a positive and happy experience they have had, continuously for 20 min, for three consecutive days. Participants will be given instructions prior to beginning their writing that will encourage them to “really let go and explore your very deepest emotions and thoughts”. Within these instructions there will be several prompts as to the kinds of experiences that they may like to write about; for example “You might link your writing to your future and who you would like to become, to who you were in the past, or to who you are now. Perhaps from being in love or being a parent, from personal achievements at work or in a hobby, or from listening to music or suddenly “being hit” by a book or painting. These are only suggestions however – you may write about any positive and happy experience(s) you like.”

Participants will also be advised that spelling and grammar are not important and that if they run out of things to write before the 20 min has ended, they should repeat what they have already written. The writing can be completed at a time and place that is convenient for the participant. It is hypothesised that by setting aside some time to concentrate on a positive experience, and embody positive emotions, this will improve the caregivers’ wellbeing and mood.

### Control conditions

#### Writing Control task (WC)

In previous trials of PWD, and WED more generally, participants allocated to the Writing Control task are often asked to write about time management [28], as this is assumed to be a neutral topic. However, in a previous pilot trial with caregivers, this control condition was found to be inappropriate because most caregivers spent a significant amount of their time caring, so it tends to be an emotionally charged topic [23]. An alternative writing control task has been developed for use with caregivers, whereby caregivers were asked to objectively describe images of outdoor scenes [24]. However, our Caring for Caregivers (C4C) Lived Experience Advisory Panel (LEAP) of caregivers felt that writing about an image of outdoor scenes was not neutral—as it could either improve mood because the image is aesthetically pleasing or it could worsen mood because many caregivers are unable to visit such places due to the demands of their caregiving responsibilities. Being attentive to this feedback, we have selected, with the help of our C4C LEAP, a set of images that are primarily used within change detection research; the images depict different rooms within a house [29]. These images not only were considered to be both neutral but also have sufficient detail to keep participants engaged enough to write about them for 20 min.

Participants will be asked to write about one image during each writing session; they will be asked to write continuously for 20 min on each of three consecutive days (the same duration as the PWD group). The writing instructions will prompt participants to avoid including any emotions in their writing, i.e. “Please do not add personal information or give your opinions on the images. Please try to be as objective and detailed as possible.” Similar to the PWD group, participants in this group will be informed not to worry about spelling or grammar and that they can complete the writing when and where is most convenient for them. If they run out of things to write before the 20 min is up, they will be instructed to repeat what they have already written.

#### Non-Writing Control group (NWC)

The participants who are allocated to the NWC condition will not be asked to complete any writing—these participants will be asked to continue with their daily activities as usual. The inclusion of the NWC is based upon feedback from the C4C LEAP that having permission to take time out of your day to write, regardless of the topic, may be beneficial. The inclusion of the NWC group will allow us to determine whether it is the act of writing or the topic being written about that produces benefits (if any).

### Measures

This study will assess the use the Positive and Negative Affect Scale (PANAS) [30] as the primary outcome for the main trial. The PANAS is made up on 20 items—10 measuring positive emotion and 10 measuring negative emotion. The positive and negative emotion scales will be analysed separately and treated as co-primary outcomes. Each item asks participants to rate the extent to which they have felt this emotion over the previous week on a 5-point Likert scale (from very slightly or not at all, to extremely). The PANAS has strong internal consistency (Cronbach’s *α* > .84) [30]. We will also measure the following potential secondary outcomes:

1. The Depression Anxiety and Stress Scale (DASS-21) [31] measures symptoms of depression, anxiety, and stress in both clinical and nonclinical samples over the past week. Each scale is comprised of seven items. The measure has strong internal consistency (Cronbach’s *α* = .88) [32].
2. Caregiver Wellbeing and Support Scale (CWSv2) [33] is a measure of wellbeing, over the past 4 weeks, that is specific to caregivers. The questionnaire was commissioned by the National Institute for Health Research (NIHR) Service Delivery and Organisation (SDO) program. It is made up of 32 items and has strong internal consistency (Cronbach’s *α* = .96) [33].
3. General Self-Efficacy Scale (GSES) [34] has one scale made up of 10 items. The questionnaire measures global self-efficacy over the past week, i.e. not in relation to any specific domain. The GSES has strong internal consistency (Cronbach’s *α* > .86) [35].
4. Leisure Time Satisfaction (LTS) measure [36] was developed specifically for caregivers to measure their engagement in pleasurable activities outside of their caring responsibilities over the past month. The scale is made up of 6 items and has strong internal consistency (Cronbach’s *α* = .94) [37].
5. Measures of overall mood will be collected both pre- and post-writing for each day of writing. Mood will be measured using a visual analogue scale (VAS) ranging from feeling very negative (1) to very positive (10).

We will also collect data on the Toronto Alexithymia Scale (TAS 20) [38]. This questionnaire measures trait alexithymia (the ability to identify emotions). As alexithymia is considered a trait characteristic, we will only administer this measure at baseline (T0). The questionnaire has three sub-scales: difficulty describing feelings, difficulty identifying feelings, and externally orientated thinking. These sub-scales are comprised of five, seven, and eight items respectively. The questionnaire has 20 items in total. The scale has good internal consistency (Cronbach’s *α* > .70) [39]. This measure will be entered as a moderator on the primary outcome within the definitive trial, in line with recent research findings that PWD is more effective for those low in alexithymia [24].

We will collect the participants’ demographic information. As part of this, we will collect data on the number of sick days (i.e. days when unable to work or complete usual daily activities because of health) and GP visits that participants have experienced at each time point. This data can then be used to measure healthcare utilisation. We will measure quality of life using the ED-5D-5L health questionnaire [40]. This measure has five items and one visual analogue scale. An index score can also be computed for use within health economics. The measure has strong internal consistency (Cronbach’s *α* = .85) [41].

For participants that are allocated to one of the writing conditions (either PWD or WC), we will ask participants to note down the time they start and finish writing each day so that we can determine whether participants adhered to the writing task instructions. Also, all writing excerpts will be assessed using the Linguistic Inquiry and Word Count (LIWC) [42]; this computer software will measure the frequency of different word classes, as well as the total number of words used. The results of the LIWC will be used to assess the validity of our manipulation, i.e. those in the PWD condition should use more positive emotion words than the WC group.

The primary outcome and secondary outcomes 1 to 4 will be collected at baseline (T0) and 1 (T1), 3 (T2), and 6 (T3) months post baseline. The primary comparison across all outcomes will be PWD versus the WC. Assessments will be completed at a location that is convenient for the participant; all participants will be offered home visits.

This study will employ a mixed-methods approach. In addition to the quantitative measures described, all participants who complete a writing task (either PWD or WC groups) will be invited to complete an exit interview. The interview discussion guide is based upon the Change Interview protocol [43], with some minor modification requested by our C4C LEAP. The aim of the interview is to understand participants’ experience of the writing tasks and the study, as well as to identify if they experienced any changes over the course of the study and what these changes could be attributed to.

### Data collection and storage

At T0, participants will be supported by a research assistant to complete the baseline assessment—this assessment will be completed at a time and location that is convenient for the participant. At subsequent time points (T1, T2, and T3), participants will be given the option to complete these assessments either online, over the phone, or on paper and return by post. The researcher conducting the phone interviews will be blinded to the participant’s group allocation. Any data that is collected as a hard copy will be stored in a locked filing cabinet at the sponsor’s site. All electronic data will be kept in password protected files on a university computer. Data will be anonymised wherever possible. The trial research assistant will take responsibility for entering the data. For the secondary outcome measures, a random sub-sample of 10% of the participants will have their data checked against the Case Report Forms (CRFs) by an independent researcher to assess the reliability and quality of the data entry. For the primary outcome, 100% of the data will be checked. Any discrepancies will be investigated by the research team. CMH will act as the data custodian and supervise all aspects of the data collection, storage, and entry. All data collection and storage will adhere to the Data Protection Act (1998).

### Planned analysis

The data analysis will be conducted by the trial statistician (SB). The findings of this trial will be reported in line with the CONSORT guidelines for pilot RCTs, including a PRISMA diagram as in Fig. 1. Recruitment rates will also be expressed as a percentage, reflecting the number of participants who gave consent to take part in this study relative to the total number of people who were approached to participate. Participant retention in the study at each of the assessment time points will be reported as a percentage. Wherever possible, the reason for study and intervention dropout will be recorded—this may not be possible for all cases where participants dropout from the study, as each participant reserves the right to withdraw from the study without giving any reason.

Adherence to the writing tasks (only relevant for the PWD and WC groups) will be determined by the number of writing extracts completed (should be 3), the self-reported times that participants started and finished writing (should amount to 20 min per day), and the results of the Linguistic Inquiry and Word Count (LIWC) software [42]. Comparison of the LIWC data should show that those in the PWD group and the WC group write similar numbers of words over the 3 days but that the trend will be for the PWD group to use more positive words than the WC.

As this is a pilot study, analyses will be descriptive, and statistical comparison between groups will not be made, but rather, data for each group will be presented separately in a table side by side, with 95% confidence intervals [44]. The amount of complete data for the primary outcome across all time points will be expressed as a percentage.

To our knowledge, there is no recommended Minimal Clinically Important Difference (MCID) for our primary outcome, the PANAS [30]. Therefore, one of the aims of this study is to suggest an MCID for use in future studies. This estimation will be based on the guidance of Copay et al. [45]: as the MCID is assumed to reflect a small effect size, the baseline standard deviation across the whole sample will be multiplied by 0.2 (this value represents a small effect size, as recommended by Cohen [46]).

The exit interviews with participants in the PWD and WC groups will be transcribed, removing all identifiable information. Where necessary, pseudonyms will be used. The transcripts from each interview will be analysed within QSR NVivo 11, using thematic analysis (TA) [47]. The analysis will involve research team members and the C4C LEAP. Once familiar with the content of the transcripts, we will generate initial codes that stay close to the data. These codes will be semantically clustered into sub-themes, and finally, these sub-themes will be clustered into main themes. The final thematic structure will be described and supported with illustrative quotes from the interviews. The use of TA will enable us to identify patterns of meaning within and across participants. If a definitive trial is justified, these qualitative results will be instrumental in refining the study protocol.

A definitive trial will be justified if (1) the primary outcome measure is at least 80% complete, (2) attrition is no greater than 40% by T3, and (3) the trial and intervention are considered acceptable and accessible by patients.

### Public and patient involvement (PPI)

PPI has featured in every step of the research process. The research team has consulted caregivers using one-to-one interviews and focus groups to clarify the study aims and rationale. These consultations confirmed the absence of support for caregivers and their enthusiasm for writing as a potential intervention. Caregivers reported that writing interventions, like Positive Written Disclosure, could be beneficial as they address: (1) caregivers’ limited opportunities to express themselves, (2) limited availability and ability to physically attend carer support groups, and (3) the increased burden that can come from providing peer support within carer support groups.

The C4C LEAP has also been involved in designing this study. Specifically, they identified alternative approaches to recruitment (e.g. involving more third sector organisations), assisted in selecting the trial outcome measures, and selected the images that will be used in the WC group. Most significantly, the C4C LEAP identified that the act of writing, irrespective of the topic, could be therapeutic—and they subsequently proposed the inclusion of the NWC group. All of the study materials were reviewed by the C4C LEAP to ensure they were clear and accessible. The involvement of the McPin Foundation, a specialist mental health research charity that champions expertise from experience in studies, has also supported the structural development of PPI within the study.

The C4C LEAP will continue to be part of the study. Their role will be to monitor and advise on recruitment (including problem solving), support qualitative data analysis, and assist in the interpretation of the study results. At the point of dissemination, they will be involved in planning activities.

### Research governance

We have produced this protocol in line with the Standard Protocol Items: Recommendations for Interventional Trials (SPIRIT) guidelines [48] (see Additional files 1, 2 and 3). This study is funded by the Dunhill Medical Trust (R431/0715) and sponsored by the University of Sussex (BSMS/16/010/JON). Local governance approval was granted by Sussex Partnership NHS Foundation Trust. Ethical approval was granted by the North-West Lancaster Research Ethics Committee on 24/11/2016 (REC reference: 16/NW/0757).

This pilot trial will be monitored by a Trial Steering Committee, with an independent chair, and including academic, clinical, trial management, and lived experience experts, in accordance with the Medical Research Council (MRC) [49] guidelines. The trial will also be monitored by a separate Lived Experience Advisory Panel (C4C LEAP).

Adverse events and issues of risk will be addressed in line with the National Institute for Health Research Good Clinical Practice guidelines. If any adverse events occurring during the study are deemed to be severe and related to the study, then this may result in the early termination of the trial—this decision will be made by the study sponsors and Trial Steering Committee. All adverse events, whether they are related to the study or not, will be reported in the final study report.

### Dissemination

We plan to disseminate the results of this study to both academic and non-academic communities. The results will be written up for publication in a peer-reviewed academic journal, using open access routes wherever possible. Additionally, all participants that request one will receive a lay summary of the study findings. We also plan to disseminate the findings via relevant third sector organisations, events, and publications, such as carers’ centres, Rethink Mental Illness, and the McPin Foundation.

## Discussion

The results of this pilot RCT will determine whether Positive Written Disclosure is an acceptable intervention for older adult caregivers of people experiencing psychosis symptoms. The exit interviews, recruitment and retention rates, and the effect sizes of the co-primary and secondary outcomes will all be used to determine whether a definitive trial of PWD in this population is justified and feasible. If the results of this trial are promising then, with the support of the C4C LEAP, the qualitative and quantitative results can be used to refine the intervention and study protocols.

If PWD is found to be effective within a definitive trial, then this intervention has the potential to improve the wellbeing of a group of people currently neglected by healthcare services. Furthermore, the self-directed nature of PWD would mean this intervention could be implemented without incurring any significant healthcare costs.

Trial status: The trial opened for recruitment in January 2017.

## Additional files

Additional file 1: C4C trial participant consent form. (DOCX 71 kb)

Additional file 2: C4C trial participant information sheet. (DOCX 199 kb)

Additional file 3: SPIRIT checklist. (DOC 120 kb)

## Abbreviations

C4C: Caring for Caregivers
CRF: Case Report Forms
CWSv2: Caregiver Wellbeing and Support Scale
DASS-21: Depression Anxiety and Stress Scale
GP: General practitioner
GSES: General Self-Efficacy Scale
LEAP: Lived Experience Advisory Panel
LIWC: Linguistic Inquiry and Word Count
LTS: Leisure Time Satisfaction
MCID: Minimally Clinically Important Difference
MRC: Medical Research Council
NHS: National Health Service
NWC: Non-Writing Control group
PANAS: Positive and Negative Affect Scale
PPI: Public and patient involvement
PWD: Positive Written Disclosure
RCT: Randomised controlled trial
SPIRIT: Standard Protocol Items: Recommendations for Interventional Trials
TA: Thematic analysis
TAS: Toronto Alexithymia Scale
VAS: Visual analogue scales
WC: Writing Control task
WED: Written Emotional Disclosure
