# Supplementary material for: Caring for Caregivers (C4C): study protocol for a pilot feasibility randomised control trial of Positive Written Disclosure for older adult caregivers of people with psychosis
Source: Pilot Feasibility Stud. 2017 Nov 21;3:63. doi: 10.1186/s40814-017-0206-z (PMC5697360; doi:10.1186/s40814-017-0206-z)
Supplement: Supplementary file 1 — C4C trial participant consent form. (DOCX 71 kb) [file 40814_2017_206_MOESM1_ESM.docx]

**Consent Form**

**Study Title:** Caring for Caregivers (C4C) **Chief Investigator:** Dr Christina Jones

**Participant ID:** ………………………………………………………………………………………

Although we are asking that you provide consent for all the points below, this does not necessarily mean you will be asked to complete all of these study activities (i.e. not all participants will be asked to complete an interview). Please complete **two copies** of this consent form – one copy will be for your records, and one will be retained by the research team.

|  |  | *Initial Box* |
| --- | --- | --- |
| 1. | I confirm that I have read and understood the Participant Information Sheet (version 1, date: 18.07.16) for the above study, and have had the opportunity to ask questions |  |
| 2. | I understand that my participation is voluntary and that I am free to withdraw at any time, without giving any reason, and without my medical care or legal rights being affected |  |
| 3. | I understand that if I choose to withdraw that any data provided up to that point will be kept by the research team |  |
| 4. | I understand that relevant sections of my and data collected during the study, may be looked at by individuals from regulatory authorities or from the NHS Trust, where it is relevant to my taking part in this research. I give permission for these individuals to have access to my records |  |
| 5. | I understand that should I disclose anything that presents new risk to either myself or others then Trust risk procedures will be followed, which may involve the release of my personal information |  |
| 6. | I agree to complete an exit interview, that will be audio recorded, discussing my experiences of the writing tasks if I receive it; and that my direct quotes may be used in a report of the findings |  |
| 7. | I give my permission for members of the research team to screen any writing tasks that I complete as part of the above study |  |
| 8. | I agree to take part in the above study |  |
| Participant Name: ………………………………………………………………………… | | |
| Participant Signature: ………………………………………………… Date: …………… | | |
| Researcher Name: ………………………………………………………………………... | | |
| Researcher Signature: ………………………………………………… Date: …………… | | |
