# Supplementary material for: Caring for Caregivers (C4C): study protocol for a pilot feasibility randomised control trial of Positive Written Disclosure for older adult caregivers of people with psychosis
Source: Pilot Feasibility Stud. 2017 Nov 21;3:63. doi: 10.1186/s40814-017-0206-z (PMC5697360; doi:10.1186/s40814-017-0206-z)
Supplement: Supplementary file 2 — C4C trial participant information sheet. (DOCX 199 kb) [file 40814_2017_206_MOESM2_ESM.docx]

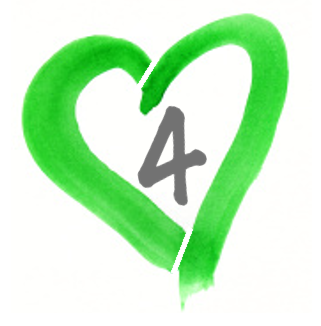

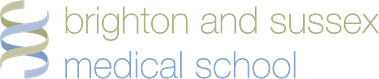
**
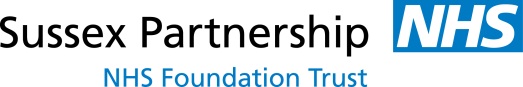
**

Caring for Carers

**Pilot randomised control trial of Positive Written Disclosure for Older Adult Carers of people with psychosis.**

Participant Information

Please take the time to read this information, discuss the study with friends and/or family, and think of any questions that you might have about the study.

You have been invited to take part in the Caring for Caregivers (C4C) study. Before you decide whether you would like to take part in this research study, it is important you read this information.

Feel free to discuss this study with anyone you would like to, and please ask the research team any questions you have regarding the research study.

**Background:**

Sometimes people experiencing psychosis need help and support to complete their daily activities. This care is mostly provided by family and friends, who tend to be in their 7-8^th^ decade. Caregiving can be both emotionally and physically demanding, and limit the caregivers ability to engage in work or leisure activities. It is important that caregivers are supported in a way that fits around their caring responsibilities.

We know that certain types of writing therapies can be helpful at reducing stress, improving wellbeing, and improving physical health for lots of different groups of people. But we do not know is this type of writing therapy will also be helpful for older adult caregivers of people experiencing psychosis. This study will help us to answer this question, and find out whether the therapy is acceptable.

**What is the purpose?**

We would like to know whether Positive Written Disclosure (PWD – a type of writing therapy) improves the wellbeing of caregivers of people with psychosis.

**Why have I been chosen?**

We have invited you to take part because we understand that you are currently providing care to someone experiencing psychosis, and are aged 60 or over.

**Are there any reasons why I wouldn’t be able to take part?**

You cannot take part if you are under the age of 60, and/or are unable to write, read and communicate in English. You cannot take part if you are not a carer, and/or provide care to someone that does not experience psychosis.

The definition of a caregiver is “any person who provides unpaid support to a partner, child, relative or friend who couldn’t manage to live independently or whose health or wellbeing would deteriorate without this help”

We have defined psychosis as anyone with a psychiatric diagnosis of schizophrenia, schizoaffective disorder, delusional disorder, psychosis, bipolar or depression with psychotic features.

Receiving this participant information leaflet does not necessarily mean that you are able to take part. If you are interested in being part of the study then we will ask you to complete a very brief eligibility assessment to determine whether this study is the right fit for you.

**Do I have to take part?**

You do not have to take part. Taking part in this study is completely up to you. You are free to withdraw from the study at any time.

**What do I have to do?**

There are a few different stages involved in this study:

If you have any questions about what you would be asked to do within this study then please contact Miss Cassie Hazell: [C.Hazell@bsms.ac.uk](mailto:C.Hazell@bsms.ac.uk)

**What are the groups that I could be allocated to?**

There are three groups that you could be randomly allocated to:

1. Positive Written Disclosure (PWD) group
2. Writing control task (WC) group
3. Non-writing control (NWC) group

The Positive Written Disclosure (PWD) group will be asked to complete the writing therapy that we think might be helpful for older adult caregivers of people with psychosis. PWD would involve you taking 20 minutes out of your day, for 3 consecutive days, to engage in some positive writing. We will send you some instructions that will guide you through the activity. We will ask you to think of a positive memory, and write about your deepest thoughts and emotions in relation to this memory.

The writing control task group (WC) will be given a series of images. If you are allocated to this group we will ask this group to take 20 minutes out of your day, for 3 consecutive days, to look at the images and write down everything that you can see in the pictures. We will ask you to be very factual and only describe what you can see.

The non-writing control group (NWC) will be asked to carry on with their usual routine and activities. We will not ask this group to complete any writing tasks.

You would be allocated to one of these three groups by someone that is independent to the research study, and does not know your name. This is done completely at random, and no one in the research team has control over the allocation of participants.

**Would I have to travel anywhere?**

Not necessarily. The research team can visit you in your home for the first meeting to fill out the forms and complete the questionnaires. If you would prefer then we can meet at an NHS facility (e.g. GP surgery or mental health building). For the questionnaires that we would like you to complete at the 1, 3 and 6 month time points, we can either send these in the post or via email for you to complete in your own time and return to us – you can also book a phone appointment so that you can complete the questionnaires with a member of our research team over the phone. If you complete an interview as part of the study, we can do this either in your home, at an NHS facility or over the phone.

**What are the drawbacks to taking part?**

- As PWD is a new therapy we do not know it if will be effective or not. We think it will help but cannot be sure.
- The study will involve you giving up some of your time to participate. The initial meeting should take no more than 1 hour (this includes filling out all the forms, talking about the study and completing the questionnaires). After this completing the questionnaires should take approximately 20-30 minutes to complete.
- Completing the writing tasks will involve you giving up some of your time to complete them. We hope that this will not cause too much of an inconvenience: we will ask you to spend 20 minutes on three consecutive days to complete them. We will support you to make a plan to complete the writing tasks. Also taking this amount of time out of your day for yourself may have beneficial effects.
- We will ask you to complete some questionnaires that ask you to think about your wellbeing and health. This could potentially cause some distress. A member of our team can support you to complete these questionnaires, and you do not have to answer any questions that you do not want to.

**What are the benefits to taking part?**

- You may have the opportunity to try a new therapy
- We will ask you to provide feedback on the study that will help shape future research studies
- You may be able to provide your opinions as to whether you think the therapy is something that should be offered to other caregivers
- All reasonable travel expenses incurred through the study will be reimbursed
- Some people have said that completing these questionnaires can offer them an opportunity to self-reflect

**Will my taking part be kept confidential?**

The only people that will know about your participation in this study will be the research team and anyone else that you choose to tell. The only time this confidentiality will be broken, is if you mention something that puts either yourself or others at risk. Under these circumstances we will have to pass on this information to your GP and any other relevant authorities.

All of the information recorded and collected during the study will be kept confidential and stored securely. The only people that will be allowed to see this are those that are part of the research team. Your name and address will be removed from all of the documents used in the study, and you will be assigned a numerical code; this code will mean that no one will know it belongs to you unless they know the code. Participants who are in the Writing Control and Positive Written Disclosure groups will be invited to take part in will be audio recorded. This recording will be transcribed (written up), and all identifiable information will be removed. The research team may use direct quotes from the interviews in a report of the study results, but these will all be completely anonymised.

The research team will securely retain your contact information for up to one year after the end of the study so that we can send you a copy of the results from this study. After this time, all records of your personal information will be securely destroyed.

All data and information collected during the course of the study will be stored at either the Sussex Partnership NHS Foundation Trust Research and Development department or the Brighton and Sussex Medical School. Any information that is kept as hard copies (i.e. paperwork) will be stored in a locked filing cabinet, and any information stored electronically (i.e. personal details) will be kept on a locked computer in password protected files. This study complies with data protection laws.

**Will anyone read what I write?**

Only the research team will read what you have written as part of either the writing control task or Positive Written Disclosure. However the researcher reading it will not know who the writing belongs to – only your participant code will be written on the writing workbook. The writing will be screened to check for any signs of severe distress or risk. If the researcher notices any issues of risk, then they will look up who the participant code refers to.

**What will happen to the results of the study?**

The results will be analysed by a member of the research team. The results of the study will be written up into a report that will hopefully be published in a scientific journal. These results may also be presented at an academic conference. You will also be asked if you would like a copy of the results when completing the consent form. We will also disseminate the study results to relevant third sector organisations (e.g. carers’ charities).

Anonymised data may also be shared with other research teams,

**Who is organising the research?**

The research is being led by Brighton and Sussex Medical School, and sponsored by the University of Sussex. The study has funding from the Dunhill Medical Trust.

**Who has reviewed this study?**

This study has been reviewed by an NHS ethics committee called North West – Lancaster Research Ethics Committee, as well as the Brighton and Sussex Medical School ethics committee.

**Who do I contact if I want to know more about this study?**

If you wish to know more about this study in particular or the research program then please email Miss Cassie Hazell: [C.Hazell@bsms.ac.uk](mailto:C.Hazell@bsms.ac.uk)

**What if something goes wrong with this study?**

It is very unlikely that there will be any adverse events associated with taking part in this research study. However if you are harmed by taking part in this research study, there are no special compensation arrangements. If you are harmed by someone’s negligence, then you may have grounds for a legal action, but you may have to pay for it.

If you would like to make a complaint regarding your treatment and participation in this study then please contact Caroline Brooks at the Brighton and Sussex Medical School on [c.e.brooks@bsms.ac.uk](mailto:c.e.brooks@bsms.ac.uk) or 01273 641470. Or contact Taffy Bakasa at the Research and Development department within Sussex Partnership NHS Foundation Trust on [Taffy.Bakasa@sussexpartnership.nhs.uk](mailto:Taffy.Bakasa@sussexpartnership.nhs.uk) or 01273 265909. The Brighton and Sussex Medical School has insurance in place to cover legal liabilities in the unlikely event that harm should arise from this study.

You may also wish to seek advice or use the more general NHS complaints mechanisms: The Patient Advice and Liaison Service (PALS) on 01323 446042 or email: [pals@sussexpartnership.nhs.uk](mailto:pals@sussexpartnership.nhs.uk).

**What happens next?**

We would like you to take at least 24 hours to think about this study and if you would like to take part. If you decided you do want to take part you will be invited to attend a consent meeting. During this meeting you will be asked to complete a consent form. This form will act as a record to show that you have agreed to take part in this study. Even once you have signed this form, you can still decide to withdraw from the research study.

If you have any further questions then please do not hesitate to get in touch with the research team.
